# Functional and Vascular Outcomes of Posterior Acetabular Wall Osteosynthesis via the Kocher–Langenbeck Approach: A Dynamic Analysis

**DOI:** 10.3390/jcm14217749

**Published:** 2025-10-31

**Authors:** Yuriy Prudnikov

**Affiliations:** Collegium Medicum, WSB University, 1C Zygmunta Cieplaka Str, 41-300 Dąbrowa Górnicza, Poland; yuriy.prudnikov@wsb.edu.pl

**Keywords:** acetabular fracture, Kocher–Langenbeck approach, open reduction and internal fixation (ORIF), peripheral circulation, neuromuscular function, electromyography (EMG), chronaximetry, rheovasography, microcirculation impairment, functional outcome

## Abstract

**Background/Objectives**: The Kocher–Langenbeck approach is widely used for surgical fixation of posterior acetabular wall fractures. While previous studies have focused on mechanical outcomes and the risk of post-traumatic osteoarthritis, the effects on peripheral circulation and neuromuscular recovery remain underexplored. This study aimed to evaluate dynamic changes in neuromuscular function and microcirculation following open reduction and internal fixation (ORIF) using this approach. **Methods**: A retrospective analysis was conducted on 34 patients (aged 23–75) treated for posterior acetabular wall fractures between 2014 and 2022. All patients underwent ORIF via the Kocher–Langenbeck approach. Assessments at 8 and 12 months postoperatively included electromyography (EMG), chronaximetry, and rheovasography (RVG). Asymmetry coefficients were calculated to quantify blood flow and functional differences. **Results**: At 12 months postoperatively, significant microcirculatory asymmetry persisted in the operated limb, with arterial and venous coefficients exceeding 25% (27.5% and 26.8%, respectively). EMG revealed sustained reductions in gluteus maximus and rectus femoris activity (asymmetry ~39%). Chronaximetry showed delayed nerve conduction recovery, particularly in the common peroneal nerve (AC = 44%). The femoral segment demonstrated the most severe impairment in both arterial inflow and venous outflow. **Conclusions**: ORIF via the Kocher–Langenbeck approach is associated with long-term disturbances in neuromuscular function and regional circulation. Further research should explore alternative surgical approaches (e.g., ilioinguinal, Stoppa) in prospective studies, assess vascular integrity using advanced imaging (e.g., contrast-enhanced ultrasound), and incorporate long-term functional outcomes. Studies on neurovascular-sparing techniques and optimised rehabilitation protocols may help reduce postoperative morbidity and improve recovery.

## 1. Introduction

Fractures of the acetabulum, particularly those involving the posterior wall, represent a complex orthopaedic condition that necessitates high-precision diagnostics and a well-considered surgical strategy. Due to the anatomical complexity of the hip joint and the high functional significance of the affected region, the choice of optimal surgical approach becomes a critical factor in achieving a successful clinical outcome. Among the available osteosynthesis techniques, the posterior Kocher–Langenbeck approach is widely adopted, owing to its favourable visual exposure of the posterior column and wall, as well as the ability to achieve stable fragment fixation. It has been described as “the workhorse for the reduction and fixation of hip fractures that require fixation via a posterior approach” by several authors [1,2], emphasising its enduring role in acetabular surgery. In addition, its anatomical rationale, technical steps, and specific indications have been comprehensively reviewed in the recent literature, underlining its continued relevance in orthopaedic trauma care [3].

The efficacy of this approach has been demonstrated in a number of studies that assessed clinical, functional, and radiological outcomes following surgical management of acetabular fractures. In particular, a retrospective study by D. Kumar et al. [4] established that the quality of reduction and fracture type exert a significant influence on both functional and radiographic outcomes. Nonetheless, even in cases of satisfactory postoperative recovery, complications are frequently observed, including the development of post-traumatic osteoarthritis and other treatment-related impairments.

Other authors have highlighted that advancements in technical aspects—such as the introduction of preoperative 3D modelling—can improve reduction accuracy, shorten the duration of surgery, and reduce intraoperative blood loss. However, as demonstrated in a randomised study by M. Bouabdellah et al. [5], such measures do not result in statistically significant improvements in long-term functional outcomes. This suggests that despite technological progress in fixation techniques, the focus continues to be placed on mechanical restoration parameters.

Although the Kocher–Langenbeck approach in the studied cohort was associated with favourable intraoperative parameters—namely, reduced operative time and moderate blood loss—these markers of technical efficiency did not result in statistically significant improvements in functional recovery. A similar observation was reported in a randomised trial comparing the modified Stoppa and ilioinguinal approaches, where the modified Stoppa approach reduced intraoperative blood loss and surgical time, yet produced no measurable difference in long-term clinical outcomes [6]. This reinforces the notion that functional restoration following acetabular osteosynthesis is influenced less by the surgical technique itself and more by postoperative neuromuscular adaptation, microcirculatory recovery, and the patient’s adherence to rehabilitation protocols. Even in cases of anatomically optimal fixation, the risk of persistent neural tension, local ischaemia, and delayed reinnervation may limit the extent of functional improvement over time.

Larger clinical series have underscored that, despite achieving anatomical reduction and relatively favourable short-term functional outcomes, the rate of complications following ORIF via the posterior approach remains high. For example, in the study conducted by L.L. Negrin et al. [7], which included 167 patients with high-energy fractures, anatomical reduction was achieved in 63.5% of cases; however, degenerative changes were found in 49.7% of patients, including post-traumatic osteoarthritis (21.6%) and avascular necrosis of the femoral head (5.4%). Neurological complications were observed in 15% of cases, and 6% of patients required revision surgery.

Comparable conclusions are drawn in the meta-analysis by P.V. Giannoudis et al. [8], which included data from 3670 cases of operatively treated acetabular fractures involving various surgical approaches: osteoarthritis developed in approximately 20% of patients following surgical treatment, while avascular necrosis and heterotopic ossification occurred in fewer than 10%. These complication rates reflect pooled outcomes across different operative techniques rather than being specific to the posterior approach. The analysis emphasised the importance of controllable factors—such as the quality of reduction, surgical approach, and timing of intervention—in shaping the overall functional outcome.

Several fundamental reviews stress that anatomical reduction remains the cornerstone in the treatment of intra-articular fractures of the lower limb, including acetabular fractures [9]. Nevertheless, anatomical reduction is often challenging due to the technical complexity of the approach, large fracture fragments, severe associated trauma, and the deep location of the joint. These factors substantially contribute to the risk of long-term disability, even when open techniques are employed.

Long-term surgical outcomes are directly dependent on the accuracy of reduction, as demonstrated in the classical study by J.M. Matta [10], in which anatomical reduction was achieved in 71% of cases and was associated with a high percentage of good and excellent functional outcomes. However, even in cases of successful reduction, complications such as osteonecrosis (3%) and femoral head wear (5%) were reported, occasionally necessitating total hip arthroplasty.

Therefore, even when high-quality anatomical reduction is achieved, the risk of late complications—such as osteonecrosis, post-traumatic osteoarthritis, and functional decompensation—remains. It is essential to recognise that such complications carry not only clinical and functional implications but also systemic consequences that extend beyond individual patient management, including long-term disability and limitations in social reintegration.

Contemporary research underscores that the state of national healthcare systems—including the effectiveness of medical care—directly influences key demographic and economic indicators such as life expectancy, mortality, and resilience to systemic crises. In the context of the COVID-19 pandemic and ongoing military conflicts, the importance of public health as a pillar of macroeconomic stability has significantly increased. Within this framework, improving the effectiveness of surgical treatment and rehabilitative medicine should be viewed not only as a clinical objective but also as a strategic component of the socioeconomic stability of regions and nations [11,12,13].

In light of the above, there is an evident need for a comprehensive approach to assessing the outcomes of posterior acetabular wall osteosynthesis. This approach should incorporate not only radiological and clinical-functional indicators, but also neurophysiological and microcirculatory parameters, which play a critical role in the long-term restoration of function. While radiographic imaging allows assessment of anatomical reduction, it does not capture the state of neural integrity, perfusion, or muscular reinnervation, all of which can substantially influence rehabilitation potential. Thus, incorporating these parameters into postoperative monitoring may offer a more complete picture of recovery and enable earlier detection of functional deficits.

The present study aims to investigate the dynamics of vascular and neuromuscular changes following open osteosynthesis using the Kocher–Langenbeck approach.

The article is structured as follows: Section 2 presents a literature review with visualisation of scientific trends based on bibliometric analysis; Section 3 describes the materials and methods, including a retrospective analysis of clinical data; Section 4 outlines the primary findings of neurophysiological and microcirculatory monitoring; Section 5 discusses these findings within the context of existing literature; Section 6 addresses the methodological and clinical limitations of the study; and Section 7 summarises the conclusions and proposes directions for future research.

## 2. Literature Review

Contemporary challenges in restorative traumatology necessitate a re-evaluation of the approaches used to assess the effectiveness of surgical treatment, particularly in relation to complex intra-articular injuries such as acetabular fractures. In this context, systematic reviews of the scientific literature are of particular importance, as they allow for the synthesis of existing evidence, identification of established research directions, and recognition of underexplored areas.

Given the above, a structured scientific analysis of publications focusing on surgical management of acetabular fractures via the posterior approach becomes especially relevant. To this end, a systematic literature search was conducted using the PRISMA methodology in order to identify prevailing scientific trends, research gaps, and directions for future investigation [14].

**Figure 1:**
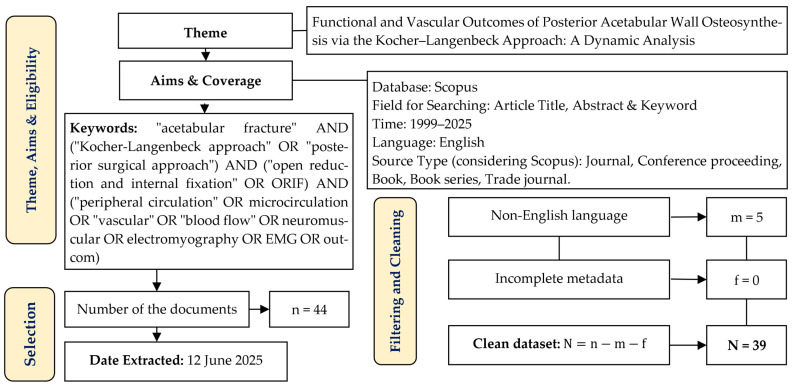
PRISMA 2020 Flow Diagram for the Identification and Selection of Studies Related to the Functional and Vascular Outcomes Following the Kocher–Langenbeck Approach.

Figure 1 presents the PRISMA 2020 flow diagram outlining the selection and screening process of the publications. The thematic scope encompassed studies addressing open reduction and internal fixation (ORIF) of posterior acetabular wall fractures using the Kocher–Langenbeck approach, with particular emphasis on functional, neuromuscular, and vascular outcomes. The literature search was conducted in the Scopus database, covering the period from 1999 to 2025, and employed keywords reflecting the anatomical target, surgical technique, and physiological parameters of recovery.

A total of 44 publications were initially identified, of which five were excluded based on language criteria (non-English), while none were excluded due to incomplete metadata. As a result, the final dataset for analysis comprised 39 articles. All selected publications were reviewed manually without the use of automated pre-sorting tools. The most relevant works—those closely aligned with the scope of the present study—were selected for detailed literature analysis.

To further identify key concepts, thematic clusters, and research trends, a visual analysis of term co-occurrence was conducted using the VOSviewer 1.6.20 software [15], based on the query “Kocher–Langenbeck approach” in the Scopus database (n = 334).

**Figure 2:**
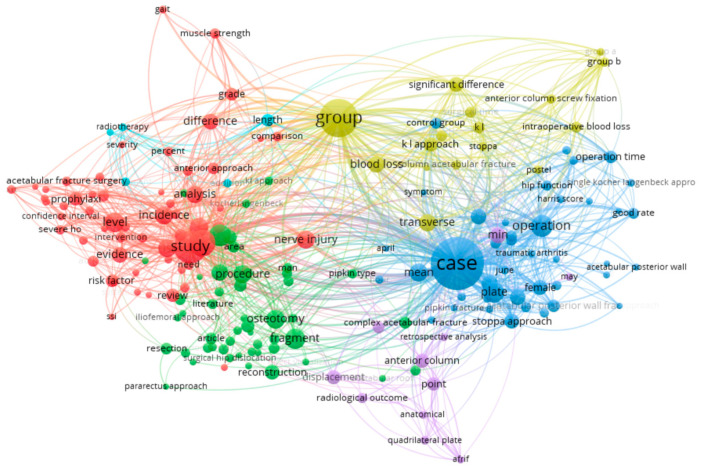
Cluster Map of Terms Associated with the Kocher–Langenbeck Approach Based on Publication Analysis in the Scopus Database (VOSviewer).

Figure 2 presents a cluster map of terms automatically generated from the analysis of 334 scientific publications retrieved with the keyword “Kocher–Langenbeck approach” using VOSviewer. The visualisation reflects the frequency of co-occurrence of terms in article titles and abstracts, allowing for the identification of thematic areas within the research domain. Each coloured cluster groups together terminologically related concepts, representing stable areas of scientific interest. In total, six thematic clusters were identified, each representing a distinct research priority.

The blue cluster (clinical outcomes & surgical technique) includes terms such as case, operation, plate, transverse, anterior column, acetabular posterior wall, min, operation time, female, and good rate. This thematic group reflects clinical and surgical aspects of treatment, including evaluation of operative time, fracture type, surgical approach, and functional outcomes—often discussed in a gender-specific context. Conclusion: this cluster highlights the practical aspects of open osteosynthesis and its clinical effectiveness.

The red cluster (complications & evidence base) comprises terms such as study, evidence, prophylaxis, level, incidence, risk factor, confidence interval, analysis, and severity. The thematic focus concerns the frequency and nature of complications (e.g., infection, heterotopic ossification), preventive strategies, and the meta-analytical approach. Conclusion: the emphasis is on evidence-based medicine and risk assessment.

The green cluster (surgical techniques & anatomy) includes terms such as procedure, fragment, reconstruction, osteotomy, nerve injury, dislocation, surgical hip dislocation, and approach. This thematic area examines variations in surgical approaches, anatomical features, and neurological complications. Conclusion: the focus is on the technical complexity of interventions.

The yellow cluster (study design & group comparisons) includes group, blood loss, significant difference, k − l approach, control group, comparison, and symptom. It deals with clinical trial structures, perioperative blood loss, and intergroup statistical significance. Conclusion: the cluster reflects a predominance of controlled studies with an emphasis on design and statistical analysis.

The purple cluster (radiographic outcomes & anatomical reduction) includes point, radiological outcome, displacement, anatomical, and quadrilateral plate. The thematic emphasis is on the precision of anatomical reduction and its radiological confirmation. Conclusion: the cluster covers visual and morphological criteria for assessing surgical results.

The small pink cluster (functional outcomes) includes gait, muscle strength, and grade. This thematic group refers to functional recovery indicators, such as gait and muscle strength. Conclusion: despite being underrepresented, the cluster highlights the need for further research into rehabilitation outcomes.

The results of the visual analysis indicate that the primary focus of the scientific community is concentrated on clinical effectiveness, the structure of complications, and the precision of surgical reduction in the treatment of acetabular fractures using the Kocher–Langenbeck posterior approach. In contrast, clusters related to neuromuscular and vascular outcomes—such as nerve injury, blood flow, and muscle strength—remain fragmented and underdeveloped. This highlights an existing research gap and underscores the relevance of the present study’s focus on these specific aspects.

**Figure 3:**
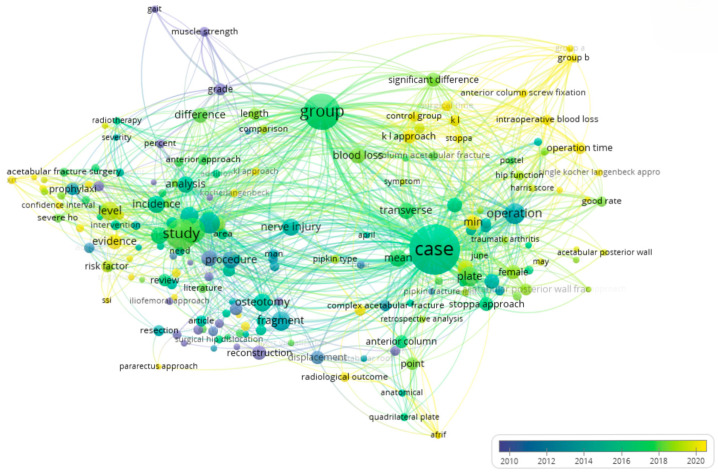
Overlay Visualisation of Keyword Co-occurrence Network Based on 334 Publications on the “Kocher–Langenbeck Approach” (VOSviewer Analysis, 1999–2025).

Further confirmation of this is provided by Figure 3, which presents an overlay visualisation of terms generated from the analysis of the same 334 publications. The colour scale of the visualisation reflects the chronological progression—from earlier studies (represented in purple/blue hues) to more recent ones (in yellow). As seen in the diagram, key areas related to clinical aspects—such as operation, plate, case, group, and significant difference—have received consistent attention in recent years. In contrast, terms reflecting vascular and neuromuscular components—such as nerve injury, muscle strength, and gait—remain within the blue and purple ranges, indicating their lower prominence or underrepresentation in more recent literature.

Thus, the overlay analysis complements the cluster structure by revealing not only thematic domains but also their evolution over time. This further emphasises the need for in-depth investigation into the functional and microvascular outcomes of surgical treatment, particularly in the context of current demands for personalised and restorative medicine.

**Figure 4:**
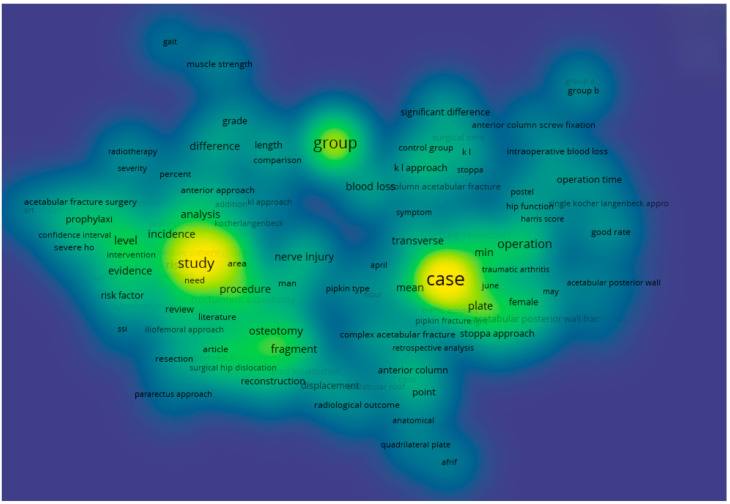
Density Visualisation of Terminological Landscape Based on 334 Publications Related to the “Kocher–Langenbeck Approach” (VOSviewer analysis).

In addition, a density visualisation of the terminological field was conducted and is presented in Figure 4. In this model, colour intensity reflects the density of scientific attention to specific terms—that is, the number and frequency of their mentions within the analysed body of literature. Bright yellow areas indicate the most actively studied concepts, while blue and purple zones suggest fragmentation and relatively low research demand.

The densest areas of the map are concentrated around the terms case, group, study, operation, plate, blood loss, and significant difference, which once again confirms the dominance of topics related to clinical outcomes, study design, and statistical interpretation. In contrast, terms pertaining to neurophysiological and vascular aspects—such as muscle strength, gait, nerve injury, and radiological outcome—are located in low-density zones, suggesting a possible underappreciation of these areas in the current scientific agenda.

Thus, the density analysis provides additional insight into current scientific priorities and allows for a more objective justification of the relevance of further research focused on neuromuscular and microvascular outcomes following acetabular osteosynthesis via the posterior approach.

In light of the findings from the visual analysis, there is a clear need for more in-depth examination of primary sources addressing clinical outcomes, neuromuscular and vascular complications, as well as functional rehabilitation following posterior wall acetabular osteosynthesis using the Kocher–Langenbeck approach.

The thematic review presented below summarises key publications that explore the role of surgical approach, patient positioning, complication development, and long-term functional outcomes, including muscle strength recovery, nerve conduction, and regional blood flow alterations.

Recent publications have placed increasing emphasis on the technical details of posterior wall osteosynthesis, including patient positioning and the minimisation of iatrogenic trauma to soft tissues, vessels, and neural structures.

For example, in a study by M. Salameh et al. [16], the impact of patient positioning (lateral versus prone) during Kocher–Langenbeck approach osteosynthesis was analysed. The results of this retrospective analysis of 73 patients showed that, while the quality of reduction did not differ significantly between groups, the lateral position was associated with a shorter operative time and a reduced incidence of iatrogenic sciatic nerve injury. At the same time, no significant differences were observed in terms of blood loss, infectious complications, or the frequency of heterotopic ossification. These findings suggest that the choice of patient position primarily affects intraoperative risks without substantially influencing functional recovery parameters.

The study by J. Reátiga Aguilar et al. [17] introduced a modified “rotator-sparing” Kocher–Langenbeck approach using spring plates, aimed at minimising soft tissue trauma while maintaining fixation stability. In the observed cohort of 24 patients with isolated posterior wall acetabular fractures, the authors reported excellent functional outcomes in 95.8% of cases at 12-month follow-up. Only one patient developed post-traumatic osteoarthritis, associated with avascular necrosis of the femoral head. These findings support the importance of tissue-preserving fixation techniques; however, the study did not directly assess neuromuscular or vascular status, limiting the comprehensive interpretation of rehabilitation potential.

A comparative analysis of patient positioning conducted by L.L. Negrin et al. [18] confirmed the absence of significant differences in the quality of reduction between patients operated in the prone versus lateral positions. However, the prone group exhibited a significantly higher rate of infectious complications (*p* = 0.017) and revision surgeries (*p* = 0.009). The authors attributed these outcomes to a higher prevalence of severe and multi-fragmentary fractures in the prone group, which were associated with longer preoperative delays and an increased risk of nosocomial colonisation. Although neuromuscular and vascular outcomes were not directly evaluated, the study indirectly suggests that trauma severity and preoperative conditions may influence the overall postoperative physiological burden.

The impact of patient positioning on clinical outcomes in pelvic fracture osteosynthesis has received growing attention in the literature. In addition to the previously mentioned studies, the work by L.L. Negrin and D. Seligson [19] offered a focused analysis of transverse acetabular fractures treated in both prone and lateral positions. Results from the retrospective review of 27 cases demonstrated that patients operated in the lateral position had significantly poorer reduction quality (*p* = 0.032) and a higher rate of post-traumatic osteoarthritis (*p* = 0.049). Notably, both instances of iatrogenic nerve injury were also recorded in this group. The authors concluded that potential biomechanical limitations associated with the lateral position may hinder adequate fragment repositioning. These findings highlight that patient positioning should be considered not only from a technical standpoint but also from a rehabilitative perspective.

The comprehensive review by P.M. Rommens [20] serves as an authoritative guide to the Kocher–Langenbeck approach, detailing the indications, contraindications, and technical execution. The study outlines the anatomical rationale for the approach, including manipulation of the musculature and posterior pelvic structures, as well as fixation techniques. In the clinical section, outcomes are reported for 60 patients with posterior wall fractures. Among them, 11.6% presented with primary neurological deficits and 8.3% developed secondary deficits. The revision rate was also 8.3%. Despite a high incidence of combined injuries, 69.6% of patients achieved excellent or good outcomes according to the Merle d’Aubigné–Postel scoring system. This source highlights both the therapeutic potential of the posterior approach and its associated neurovascular risks, thereby reinforcing the relevance of the present study.

Of particular interest is the study by I.A. Yavuz et al. [21], which examined the impact of surgical intervention for isolated acetabular fractures on sexual function in patients and their partners. This prospective study included 65 patients who underwent osteosynthesis via one of three approaches: Kocher–Langenbeck, ilioinguinal, or a modified Stoppa approach. Based on assessments using internationally validated scales, male patients in the posterior approach group experienced the least reduction in erectile function. Among female patients, no significant differences were observed between groups. This finding indirectly suggests reduced disruption to pelvic neurovascular structures when using the posterior approach, offering an additional rationale for further investigation of its neurovascular preservation potential.

Minimally invasive techniques in acetabular fracture management are increasingly adopted in clinical practice. M. Qoreishy et al. [22] described a hybrid approach combining the posterior Kocher–Langenbeck technique with anterior percutaneous osteosynthesis. Among 155 patients, a high rate of anatomical reduction (74.8%) and excellent functional outcomes (75.5% according to the Harris Hip Score) were achieved, with a low complication rate. This method demonstrated reliability and efficacy while maintaining a minimally invasive profile.

The issue of transfusion support remains clinically relevant. A. Jawed et al. [23] compared 119 patients who underwent surgery with and without intraoperative cell salvage (CS). No significant differences were found in the volume or frequency of allogeneic transfusions, regardless of the surgical approach used. In cases of severe trauma (ISS > 20), transfusion volumes were elevated irrespective of CS use. The authors do not recommend routine implementation of cell salvage techniques in pelvic surgery.

J. Borrelli et al. [24] demonstrated the importance of muscle strength restoration for functional outcomes following osteosynthesis. Although the study primarily focused on the anterior approach, it revealed a correlation between reduced strength—particularly of the hip flexors and adductors—and lower scores on the MFA questionnaire. Radiographic findings and range of motion did not correlate with overall functional status. The authors emphasised the need for standardised evaluation protocols and structured rehabilitation programmes.

The analysis of neurovascular complications and functional recovery following Kocher–Langenbeck osteosynthesis warrants particular attention. G.J. Haidukewych et al. [25] showed that intraoperative neurophysiological monitoring does not reduce the incidence of iatrogenic sciatic nerve injuries. In their study involving 252 cases, the rate of nerve injury was paradoxically higher in the monitoring group (8.9% vs. 2.9%), and in most instances, the monitoring system failed to detect abnormalities. These findings highlight the complexity of preventing neurological complications and the need for additional risk predictors.

Further insights into long-term functional outcomes are provided by another study by J. Borrelli et al. [26], which involved a comprehensive assessment of 15 patients with isolated acetabular fractures treated via the posterior approach. Although muscle strength and gait parameters were restored to normal, some patients experienced reduced quality of life as measured by the MFA scale. A significant decline in hip flexor and extensor strength was associated with poorer functional recovery, whereas radiographic and biomechanical indicators showed no meaningful differences. This underscores the importance of objectively assessing neuromuscular status and its role in outcome prediction.

In contrast to the abundance of studies focused on mechanical stability, anatomical reduction, and radiographic results, the dynamics of neuromuscular function and regional microcirculation following posterior acetabular osteosynthesis remain significantly underexplored. The present study aims to address this gap by shifting the emphasis from purely structural outcomes to functional and physiological recovery parameters.

In summary, the current literature review has identified substantial gaps in the study of neuromuscular and microvascular recovery dynamics following posterior wall acetabular osteosynthesis via the Kocher–Langenbeck approach. Despite the accumulation of data concerning surgical technique and complication rates, systematic analysis of functional and haemodynamic parameters—reflecting the quality of neuromuscular recovery and regional blood flow—remains highly limited. These findings emphasise the necessity of a more in-depth clinical and physiological approach capable of evaluating not only anatomical but also functional repair.

In this context, the aim of the present study is to conduct a comprehensive assessment of the dynamics of functional changes in the neuromuscular system and regional microcirculation in patients following open osteosynthesis of the posterior acetabular wall performed using the Kocher–Langenbeck posterior surgical approach.

To achieve this objective, a staged assessment was implemented, involving the registration of electromyography (EMG), chronaximetry, and rheovasography (RVG) parameters at various postoperative intervals. This approach enabled the monitoring of both neuromuscular function recovery and circulatory parameters in the area of surgical intervention, thereby providing a more complete understanding of the clinical consequences of the treatment.

A detailed description of the methods employed, patient inclusion criteria, and characteristics of the functional tests performed is provided in the following section.

## 3. Materials and Methods

As part of a retrospective cohort study, data from 34 patients with posterior wall acetabular fractures treated at the Institute of Traumatology and Orthopaedics in Lyman, Ukraine, between 2014 and 2022 were analysed. All patients underwent surgical treatment by means of open osteosynthesis using the standard Kocher–Langenbeck posterior approach, followed by a structured rehabilitation programme.

The study included patients aged between 23 and 75 years. According to anamnesis, the primary causes of injury were road traffic accidents (25 patients), falls from height (4 patients), and compression trauma resulting from structural collapse (5 patients). All injuries were the result of high-energy trauma, which is known to be associated with complex fracture morphology and an increased risk of concomitant soft tissue damage. Based on the fracture pattern, 22 patients were diagnosed with monofragmentary fractures, and 12 with comminuted fractures. In 27 cases, the fracture was accompanied by dislocation of the femoral head. Exclusion criteria included low-energy trauma (e.g., domestic falls), and previous surgery to the affected hip. These criteria were applied to ensure a homogeneous study group with comparable injury mechanisms and treatment indications.

In addition, patients with pre-existing peripheral neuropathies, radiculopathies, disorders of neuromuscular transmission (e.g., myasthenia gravis), systemic vasculitis, significant peripheral arterial disease, or prior trauma and surgical interventions on the same limb were excluded from the study. Only isolated fractures of the posterior wall of the acetabulum were included, classified as types A1 and A2 according to the AO/OTA system. Patients with associated or combined fracture patterns were not considered for inclusion.

The timing of surgery varied: 26 patients underwent operative treatment within 6–12 days post-injury, 6 patients between 13–21 days, and 2 patients one month or more after the trauma. The surgical procedure included open reduction and fixation of fracture fragments using screws or plates. The timing of surgery varied: 26 patients underwent operative treatment within 6–12 days post-injury, 6 patients between 13–21 days, and 2 patients one month or more after the trauma. The surgical procedure included open reduction and fixation of fracture fragments using screws or plates.

Delayed osteosynthesis was performed due to the late admission of patients transferred from other hospitals, during the active phase of the post-traumatic disease period, when clinical and laboratory findings were not favourable for surgery and the procedure had to be postponed until their condition stabilised. In addition, there were four cases with delayed surgical intervention due to extensive skin abrasions in the planned surgical approach area.

In the postoperative period, 27 patients received orthopaedic management in the form of skeletal or cuff traction for 2–3 weeks. This was followed by an unloading phase—ambulation with crutches without weight-bearing on the operated limb for up to 3 months. Full weight-bearing was typically achieved by 5–6 months postoperatively. Throughout the recovery period, patients performed therapeutic exercise with progressive loading and underwent physiotherapy sessions.

Functional assessment of peripheral circulation and neuromuscular function was conducted 8 months post-surgery, during the stage of weight-bearing restoration, gait pattern re-establishment, movement coordination, and proprioceptive sensitivity recovery. A follow-up evaluation was performed at 12–13 months postoperatively.

The 8-month assessment point was selected to correspond with the stage of remyelination and microvascular remodelling, when early post-traumatic inflammation has subsided, and neuromuscular and circulatory adaptations begin to stabilise. The 12-month follow-up was intended to capture long-term outcomes and the plateau phase of functional recovery. Earlier assessments (e.g., at 3–6 months) were not conducted in order to minimise the confounding effects of acute inflammatory responses and transient functional deficits. Later evaluations were not included, as they fell outside the scope of the study’s observational window.

Rheovasographic assessment was performed using the RG-02 rheograph with four-channel oscillographic signal registration. Electrodes (surface area of 2 cm^2^) were placed along the neurovascular bundle of the examined limb, spaced 12 cm apart. Signal acquisition was conducted following a three-minute adaptation period during breath-hold at the end of expiration.

During visual analysis, the following characteristics were evaluated: wave rhythm, shape, and amplitude; the contour of the anacrotic rise and catacrotic descent; and the symmetry of waveforms between the two limbs.

Quantitative parameters included: the amplitude of the systolic wave (reflecting pulse blood filling), the time to peak systolic blood filling (indicating arterial inflow), the duration of the descending part of the waveform (characterising venous outflow), as well as the amplitudes of the dicrotic notch and diastolic wave.

Additionally, the dicrotic index (the ratio of the amplitude at the dicrotic notch to the maximum amplitude of the systolic wave) and the diastolic index (the ratio of the diastolic wave amplitude to the maximum systolic amplitude) were calculated. These indices reflect the characteristics of venous outflow and vascular tone within the microcirculatory bed.

To assess blood flow asymmetry, an asymmetry coefficient (AC) was calculated using the following formula:

AC = (1 − α/β) × 100%,(1)

where α represents the signal amplitude on the operated limb, and β denotes the corresponding value on the intact side.

Bioelectrical activity of the lower limb muscles and conduction along the tibial and peroneal nerves were recorded using an MG440 electromyograph. The following muscles were assessed: gluteus maximus, paravertebral muscles, biceps femoris, rectus femoris (as part of the quadriceps group), and the muscles of the peroneal and tibial compartments.

The inclusion of paravertebral muscles in the analysis was based on their compensatory role in trunk stabilisation during locomotion and rehabilitation. Following acetabular fractures, especially in cases of compromised gluteal function, these muscles may exhibit increased activation to maintain postural control and gait symmetry. Their assessment, therefore, provides additional insight into patterns of neuromuscular adaptation during the recovery period.

Surface EMG was performed using disposable Ag/AgCl electrodes (10 mm diameter, 20 mm interelectrode distance; Neurosoft, Ivanovo, Russia) in a bipolar configuration. Electrodes were placed over the erector spinae, gluteus medius, quadriceps femoris, tibialis anterior, and peroneus longus muscles according to SENIAM recommendations. Signals were amplified (gain = 1000), band-pass filtered between 10–500 Hz with a 50 Hz notch filter, and digitised at a sampling rate of 2000 Hz (16-bit resolution; Neuro-MEP-Micro system). Biopotentials were recorded both at rest and during maximal voluntary contraction, with amplitude measured in millivolts.

To reduce inter-individual variability, all EMG and chronaximetry recordings were normalised relative to the contralateral (unaffected) side. This approach allowed minimisation of confounding factors such as skin impedance, electrode placement, and muscle mass differences, and enabled a focus on within-subject asymmetries between the operated and intact limbs. The resulting data are presented as percentage asymmetry values, reflecting side-to-side differences rather than absolute amplitude values. Asymmetry coefficients (AC%) are presented as mean ± standard deviation, as the distribution of the data was approximately normal according to the Shapiro–Wilk test.

Statistical analysis was performed using standard methods. Descriptive statistics included the calculation of the mean (X), standard deviation (SD), and standard error of the mean (SEM). The normality of data distribution was assessed using the Shapiro–Wilk test. For comparison of paired measurements between the operated and unaffected limbs, the paired Student’s *t*-test was used for normally distributed data; otherwise, the Wilcoxon signed-rank test was applied. Correlations between variables were evaluated using Pearson’s correlation coefficient for parametric data and Spearman’s rank correlation for non-parametric data. All *p*-values were two-tailed, and values of *p* < 0.05 were considered statistically significant. Statistical analysis was conducted using licenced software; the specific version is available upon request.

All stages of the study were conducted in accordance with the Declaration of Helsinki and approved by the local ethics committee of the Institute of Traumatology and Orthopaedics (No. 12/14 dated 17 February 2014). Written informed consent was obtained from all participants. All protocols, methods, and primary data are available from the corresponding author upon request.

Following the completion of all observation, registration, and data processing stages, a final cohort was established for the analysis of neuromuscular activity and regional haemodynamics at various time points after surgical intervention. The resulting data enabled an assessment not only of the degree of functional recovery in the affected limb but also of the dynamic changes reflecting the effectiveness of the rehabilitation period.

The findings of the rheovasographic and electrophysiological assessments, along with their statistical interpretation, are presented in the following section. These data form the basis for evaluating functional recovery and analysing the dynamics of neuromuscular and vascular parameters following posterior wall acetabular osteosynthesis.

## 4. Results

Assessment of the dynamics of the asymmetry coefficient (AC) enabled the analysis of changes in the functional state of peripheral circulation and the neuromuscular system at delayed time points following surgical intervention. The results of the rheovasographic assessment are presented in Table 1.

According to the obtained data, asymmetry coefficients (AC) of regional blood flow remained elevated, often exceeding 50%, and demonstrated a tendency to increase over time, particularly in the thigh and foot regions. To provide a more detailed evaluation of these changes, Table 2 presents the results of a comparative analysis of key rheovasographic parameters in patients at 8 and 12 months following open posterior wall acetabular osteosynthesis via the Kocher–Langenbeck approach.

The rheovasographic analysis included four key indicators: the amplitude of the systolic wave (A_2_), reflecting the volume of pulse blood filling; the dicrotic index (DCI), characterising venous outflow and arteriolar tone; the diastolic index (DSI), indicative of venous return and microcirculation; and the parameter of vascular bed blood filling (BF, mm). For each indicator, values were recorded for both the intact (>) and operated (<) limbs, followed by calculation of the asymmetry coefficient (AC%), which enabled an objective evaluation of blood flow impairment across different segments of the lower limb.

At 8 months postoperatively, asymmetry coefficients remained substantial. The systolic wave amplitude (A_2_) demonstrated asymmetry ranging from 30% to 58%, most prominently in the lower leg. Both the dicrotic index (DCI) and diastolic index (DSI) indicated disturbances in venous outflow and vascular tone regulation, especially in the foot, where the AC reached 51% and 47%, respectively. Blood filling (BF) parameters showed asymmetry ranging from 12% to 19%.

At 12 months and beyond following surgery, impairments persisted, and in some segments, even worsened. For example, A_2_ asymmetry increased to 50% in the foot, while DCI and DSI continued to show marked discrepancies between the healthy and operated limbs (up to 50% and 45%, respectively). Similarly, the BF parameter showed asymmetry reaching 26% in the thigh, despite the extended rehabilitation period.

Thus, the data presented in the table confirm the presence of persistent disturbances in regional blood flow and microcirculation in the long term after posterior wall acetabular osteosynthesis. The most pronounced changes were observed in the lower leg and foot segments, highlighting their vulnerability and the need for special attention during the rehabilitation process.

To further evaluate the state of regional microcirculation, a detailed analysis of rheovasographic parameters in the thigh region was conducted in patients more than 12 months after posterior wall acetabular osteosynthesis.

Table 2 presents the mean values of key parameters, including pulse blood filling amplitude (A_2_), arterial (DCI) and venous (DSI) capillary indices, and the rheographic index (RI). Each parameter is shown for both the healthy and operated limb, along with the calculated asymmetry coefficient (AC), enabling an objective evaluation of the severity of microcirculatory disturbances during the late postoperative period.

Data analysis revealed persistent circulatory impairments, most pronounced in the femoral segment. The asymmetry coefficients for the arterial and venous capillary indices were 27.5% and 26.8%, respectively, which substantially exceed the reference range for physiological asymmetry reported in healthy individuals (mean AC = 13.54% ± 4.41%) according to S.V. Sedochenko and N.P. Grachev [27]. These findings indicate sustained arterial insufficiency and impaired venous outflow. The amplitude of pulse blood filling (A_2_) also demonstrated marked asymmetry (24.3%), confirming reduced blood supply efficiency. Although the rheographic index (RI) exhibited a lower degree of asymmetry (18.1%), it nonetheless reflected ongoing functional limitations in regional microcirculation.

Similar, though less pronounced, changes were observed in the lower leg and foot: while asymmetry coefficients in these regions did not exceed critical thresholds, a persistent tendency towards impaired venous outflow was noted. This suggests that microcirculatory insufficiency is primarily localised in the femoral segment, whereas the distal regions are likely affected secondarily due to dysfunction upstream.

Importantly, the observed haemodynamic impairments showed significant correlations. Venous capillary outflow in the thigh was associated with arterial inflow (r = 0.374; *p* = 0.042); arterial and venous flow in the lower leg were closely linked (r = 0.581; *p* = 0.001); and parameters in the foot correlated not only with each other (r = 0.683; *p* = 0.001), but also with arterial perfusion in the lower leg (r = 0.466; *p* = 0.009). These results confirm the systemic nature of microcirculatory disturbances.

Thus, the data presented in Table 2 indicate that the femoral segment of the lower limb represents the most vulnerable area, where both arterial and venous microcirculatory insufficiency persist. This condition may serve as a pathogenetic basis for the development of secondary trophic disorders and degenerative changes within the joint. Despite partial stabilisation in the distal segments, full restoration of microcirculation in the thigh is not achieved, highlighting the need for targeted attention in the development of rehabilitation strategies.

To further assess the state of the neuromuscular system, a chronaximetry study was conducted. The results are presented in Table 3.

Data analysis indicates a marked tendency toward a reduction in the asymmetry coefficient (AC) over the long term. Specifically, for the peroneal nerve, the AC decreased from 59% at 8 months postoperatively to 44% at 12 months, suggesting a gradual improvement in nerve conduction, although the value remains above the physiological norm (approximately 25%).

For the tibial nerve, the changes were less pronounced: the AC was 31% at 8 months and 27% at 12 months. Despite this positive trend, residual asymmetry persists, indicating a slower recovery of neural conductivity.

Thus, the results of the chronaximetric analysis confirm that even one year after osteosynthesis of the posterior acetabular wall, functional disturbances in peripheral nerves remain. These impairments are more pronounced in the peroneal nerve, emphasising the need for comprehensive rehabilitation programmes aimed at restoring neuromuscular function.

To provide a more detailed characterisation of the neuromuscular system, an analysis of the bioelectrical activity of specific muscle groups was conducted using electromyography. The results of this investigation are presented in Table 4.

The analysis of the data presented in Table 4 reveals persistent disturbances in the bioelectrical activity of muscles in the operated limb one year after surgical intervention. The most pronounced changes were observed in the gluteus maximus muscle, where the bioelectrical activity on the non-operated side (42 ± 10.823 mV) significantly exceeded that of the operated side (25.333 ± 6.39 mV). This is likely attributable to both the initial traumatic impact and iatrogenic injury during the surgical procedure. The asymmetry coefficient (AC) for this muscle group reached 39% (±0.11), indicating a marked reduction in functional activity.

Comparable impairments were noted in the rectus femoris component of the quadriceps, as well as in the tibialis and peroneal muscle groups, with asymmetry coefficients reaching 35.8%, 37.3%, and 35.9%, respectively. These findings suggest a sustained deficit in bioelectrical activity, reflecting incomplete restoration of neuromuscular conduction even in the long term.

Although differences were less pronounced, the paravertebral and gastrocnemius muscles also demonstrated reduced signal amplitudes on the operated side. Asymmetry coefficients for these muscles remained elevated at 26.7% and 30.2%, respectively—exceeding the physiological range and indicating residual dysfunction.

Overall, the EMG data suggest that open reduction and internal fixation of the posterior acetabular wall via the Kocher–Langenbeck approach is associated with persistent functional alterations in key lower limb muscle groups. The muscles responsible for stabilising the hip joint and facilitating gait appear particularly vulnerable, which carries direct clinical implications for patients’ quality of life and functional rehabilitation. These results underscore the importance of incorporating targeted neuromuscular stimulation, resistance training, and peripheral nerve conduction recovery strategies into postoperative rehabilitation protocols.

Thus, the results of the comprehensive analysis confirmed the presence of persistent disturbances in both regional microcirculation and neuromuscular conductivity even one year after osteosynthesis of the posterior acetabular wall. The most pronounced impairments were localised in the femoral segment, where arterial inflow and venous outflow remained insufficient. Additionally, the gluteus maximus and quadriceps femoris muscles exhibited high asymmetry coefficients in bioelectrical activity. Chronaximetric data revealed delayed recovery of peripheral nerve conduction, particularly in the peroneal nerve.

Collectively, these findings reflect incomplete restoration of weight-bearing function and microcirculatory support of the limb, which may contribute to the risk of trophic disturbances and secondary degenerative changes. The results provide a foundation for a more in-depth analysis of the underlying pathophysiological mechanisms and the development of optimised rehabilitation strategies, which are discussed in detail in the following section.

## 5. Discussion

The results of the present study suggest that even when satisfactory anatomical reduction and stable fixation of posterior acetabular wall fractures are achieved using the Kocher–Langenbeck approach, persistent impairments in neuromuscular conductivity and regional microcirculation may remain detectable up to one year postoperatively.

These findings are consistent with previous research highlighting the critical role of fracture type and quality of reduction in determining radiological and functional outcomes. For instance, Kumar et al. [4] and Bouabdellah et al. [5] demonstrated that while the introduction of advanced techniques such as preoperative 3D modelling improves the precision of reduction and reduces intraoperative blood loss, it does not exert a significant impact on long-term functional results. Similarly, Negrin and Seligson [7], along with Giannoudis et al. [8], reported high rates of degenerative complications and neurological deficits even in the context of optimal reduction. The classical study by Matta [10] established a direct correlation between anatomical restoration and improved outcomes, while also noting the risk of secondary complications such as osteonecrosis and post-traumatic osteoarthritis.

The present findings build upon these observations by shifting the analytical focus from clinical and radiological outcomes to neurophysiological and haemodynamic parameters. The observed asymmetry in rheovasographic indices and muscular bioelectrical activity indicates incomplete restoration of arterial inflow, venous outflow, and peripheral nerve conduction—most notably in the gluteal, quadriceps, tibial, and peroneal muscle groups. This suggests that the sequelae of acetabular fractures extend beyond mechanical stability and affect the integrated function of neuromuscular and vascular systems.

These asymmetries carry important clinical implications. For example, EMG deviations of up to 39% in the gluteus maximus and quadriceps femoris reflect impaired muscle activation, which may contribute to hip instability, gait asymmetry, and diminished strength during weight-bearing activities. Patients with such impairments often report difficulty with stair climbing, rising from a seated position, and walking longer distances—factors that can reduce independence and quality of life.

Likewise, blood flow asymmetries exceeding 50%, particularly in the foot and lower leg, point to persistent microcirculatory deficits. These may predispose patients to local hypoxia, swelling, cold intolerance, and delayed soft tissue recovery. Such vascular insufficiency may also hinder wound healing, increase ambulation-related fatigue, and elevate the risk of secondary trophic complications. Therefore, the identified neurophysiological and haemodynamic abnormalities are not only quantifiable markers of dysfunction but also clinically meaningful contributors to long-term functional limitations.

At the same time, several studies have underscored the significance of intraoperative variables. Salameh et al. [16], for instance, demonstrated that lateral patient positioning reduces operative time and the incidence of iatrogenic sciatic nerve injury, although it does not influence long-term outcomes. Reátiga Aguilar et al. [17] introduced a modified rotator-sparing approach, which reduced soft tissue trauma and improved early functional scores. However, these investigations did not directly assess microcirculatory or neuromuscular conductivity, thereby limiting their comparability with the present findings. Negrin and colleagues [18,19] likewise noted outcome variations linked to patient positioning and fracture complexity, particularly in terms of infection rates and post-traumatic arthrosis, yet did not address neurovascular or functional parameters.

The present data are in line with the review by Rommens [20], which emphasised both the advantages of the Kocher–Langenbeck approach and the elevated risk of neurovascular complications. The observed disturbances in bioelectrical activity and the delayed recovery of neural conduction—especially involving the peroneal nerve—support the view that such complications significantly affect long-term prognosis. Notably, the study by Yavuz et al. [21], focused on postoperative sexual function, found less disruption of neurovascular structures following posterior access, indirectly supporting its advantages. Nonetheless, these findings underscore the need for more refined assessments of neurovascular outcomes.

An important consideration is the recovery of muscle strength. Studies by Borrelli et al. [24,26] have demonstrated that muscular weakness, rather than radiological parameters, correlates most strongly with patients’ quality of life. These findings are directly consistent with the present EMG results, where persistent deficits in bioelectrical activity of the gluteal and quadriceps muscles were associated with high asymmetry coefficients, indicating incomplete restoration of functional status. Similarly, the work of Engsberg et al. [28] reported gait disturbances despite adequate fracture fixation, which aligns with our observations.

Furthermore, the data on neurological complications presented by Haidukewych et al. [25] indicate the limited effectiveness of intraoperative neuromonitoring in preventing iatrogenic injuries. In our cohort, the persistence of impaired peripheral nerve conduction one year postoperatively further underscores the complexity of this issue and the need to develop new rehabilitation strategies.

Contemporary research highlights the need to optimise both surgical technique and postoperative recovery strategies. While advancements in reduction and fixation methods have improved intraoperative precision and stability, our findings suggest that achieving anatomical alignment alone may not be sufficient for full functional recovery. The persistence of microcirculatory and neuromuscular dysfunctions up to one year postoperatively underscores the importance of integrating vascular and neurophysiological assessments into long-term rehabilitation planning.

Gupta et al. [29] showed that the trochanteric flip osteotomy significantly improves visualisation of fracture fragments and reduces soft tissue damage when using the Kocher–Langenbeck approach. Their data reported no significant neurovascular complications, which contrasts with our findings of persistent functional deficits—particularly involving the peroneal nerve. This discrepancy emphasises that minimising traction-related trauma alone does not eliminate the risk of long-term conduction impairments.

On the other hand, Mears et al. [30], in a large cohort study, demonstrated that outcomes are directly dependent on initial fracture characteristics and the quality of reduction. Despite a high rate of anatomical reconstruction, a substantial proportion of patients experienced unfavourable functional outcomes, especially in the presence of concomitant injuries or in older populations. These findings are in line with our observations that, even with adequate fixation, functional results may remain limited due to the combined impact of neurological and vascular factors.

Additional research supports the relevance of personalised surgical planning. Agarwal et al. [31] proposed a virtual planning approach using the contralateral pelvic hemiring, which enabled acceleration of the modelling process and increased the precision of individually contoured plates. Such strategies appear promising in reducing operative trauma and intraoperative blood loss; however, our findings underscore the necessity of parallel assessment of not only mechanical, but also neurophysiological parameters.

Of particular interest are recent developments integrating digital technologies. In a recent review by Prudnikov et al. [32], it was demonstrated that artificial intelligence can improve diagnostic accuracy and optimise surgical decision-making in orthopaedics. In the context of our research, this opens the potential for developing algorithms for preoperative prediction of neurovascular complication risks, followed by personalised rehabilitation protocols tailored to the patient’s specific functional profile.

Thus, comparison of our results with those reported in the literature confirms the importance of a comprehensive approach that combines mechanical reconstruction with the assessment and restoration of neuromuscular and vascular functions. The next section of the analysis addresses alternative surgical strategies, limitations of the current study, and future directions aimed at improving the quality of functional rehabilitation in patients.

As an alternative to the posterior Kocher–Langenbeck approach, the literature discusses various anterior extrapelvic approaches in detail, with the ilioinguinal approach proposed by Letournel being the most prominent. Its primary advantage lies in minimising injury to the gluteal musculature and the sciatic nerve, thereby reducing the risk of major neurovascular complications. However, the limited visualisation of the posterior wall renders this approach less versatile, making it more suitable for anterior and both-column fractures.

Contemporary reviews and meta-analyses indicate that anterior approaches (including the ilioinguinal, Stoppa, and pararectus techniques) yield satisfactory functional and radiographic outcomes with comparatively reduced surgical trauma. Nonetheless, the selection of surgical access should always take into account the anatomical pattern of injury and the technical expertise of the surgeon. Notably, studies by Ramadanov et al. [33], Gänsslen et al. [34], Sallam Masoud et al. [35], as well as multicentre and comparative studies by Scrivano et al. [36], Rajnish et al. [37], Wang et al. [38], Shigemura et al. [39,40], Srivastava [41], Ciolli et al. [42], and Cao et al. [43], underscore the potential of these approaches as less invasive yet indication-specific techniques. Therefore, the ilioinguinal approach may be considered a less traumatic alternative with respect to pelvic neurovascular structures, although its application is mainly justified in cases of anterior or combined fracture localisation.

The modified Stoppa approach (anterior intrapelvic approach, AIP) holds a significant position among alternative techniques, particularly in cases involving medial fractures and anterior-central displacement. Its main advantage lies in the enhanced visualisation of the internal pelvic surface and the reduced risk of gluteal muscle and sciatic nerve injury, making it less traumatic to neurovascular structures. However, it is limited by challenges in posterior column fixation, rendering it unsuitable for isolated posterior wall fractures. According to the literature, the AIP and its modifications demonstrate high efficacy in the fixation of complex anterior acetabular fractures, offering sufficient exposure and an acceptable complication profile. Several studies [44,45,46,47,48,49] have reported that this approach enables high-precision reduction, minimises operative trauma, and reduces the risk of ischaemic complications. Nonetheless, its application requires advanced technical expertise and meticulous patient selection. In the context of the present study, the Stoppa approach may be considered a less invasive alternative in terms of neurovascular preservation, though it does not represent a universal solution for posterior wall fractures.

The trochanteric flip osteotomy (Ganz osteotomy of the greater trochanter) is another alternative to the conventional posterior approach, particularly in complex posterior and transverse fracture cases where extended exposure is required. The key advantage of this method is the ability to achieve full visualisation of the acetabulum while preserving the function of the external rotators, thereby reducing the risk of sciatic nerve injury and improving the conditions for anatomical reduction [50,51,52]. However, this method remains more invasive and is associated with risks such as non-union of the greater trochanter, damage to abductor muscles, and complications involving the femur [53,54,55]. Long-term follow-up studies have shown a high probability of satisfactory functional outcomes (up to 80% according to Steppacher et al. [56]) and reliability in treating fractures of the femoral head and posterior wall of the acetabulum [57,58]. Nevertheless, systematic reviews and comparative analyses [59,60,61] emphasise that the complication rate remains higher than with the classical Kocher–Langenbeck approach, necessitating strict patient selection and a high level of surgical skill. Thus, while the Ganz osteotomy may reduce the risk of neurovascular injury, it is associated with an increased likelihood of osseous complications, limiting its widespread use in routine clinical practice.

Minimally invasive and hybrid approaches are becoming increasingly prominent in contemporary pelvic surgery. These techniques typically involve combinations of limited anterior and posterior approaches or the utilisation of 3D printing and virtual planning technologies to optimise fixation trajectories and reduce intraoperative trauma. Recent publications demonstrate that such methods can yield outcomes comparable to those of traditional surgical approaches while reducing blood loss and shortening operative time [62,63,64,65,66]. Furthermore, robotic and navigation-assisted technologies, along with the use of patient-specific 3D-printed guides, contribute to greater accuracy in implant placement and improved prognosis by minimising the risk of technical errors. Despite these promising results, the widespread adoption of these methods is currently limited by the need for costly equipment, a steep learning curve, and a lack of robust evidence regarding long-term functional and neurovascular outcomes.

Thus, minimally invasive and hybrid strategies represent a promising direction focused on reducing surgical trauma and tailoring the operative approach to individual patient anatomy. However, their effectiveness requires further validation in prospective studies, which should include a comprehensive assessment not only of anatomical reduction but also of microcirculatory and neuromuscular parameters—an issue particularly relevant in light of the findings presented in this study.

## 6. Limitations

Several methodological and clinical limitations of the present study must be acknowledged. First, the retrospective design increases the risk of selection bias and limits the strength of causal interpretations. The relatively small sample size (n = 34) reduces statistical power and precludes stratification by fracture type, age group, or other potentially relevant variables.

Second, there was heterogeneity in the timing of surgical intervention (ranging from 6 to over 30 days post-injury), which may have influenced the dynamics of microcirculatory and neuromuscular recovery. In addition, although chronaximetry, rheovasography, and electromyography allowed for objective functional assessment, these methods are sensitive to electrode positioning, patient cooperation, and other technical variables, which may have introduced measurement variability. The absence of imaging-based vascular diagnostics—such as CT angiography, MR angiography, or contrast-enhanced ultrasound—also limited the ability to confirm microcirculatory disorders morphologically.

Third, the study lacked a control group treated using alternative surgical approaches (e.g., ilioinguinal or modified Stoppa), which restricts conclusions about the relative neurovascular safety of the Kocher–Langenbeck approach.

Fourth, although the rehabilitation protocol was standardised at the level of core principles—namely, early mobilisation and progressive weight-bearing—the intensity, frequency, and individual adherence varied across patients. This variability may have influenced functional recovery and introduced an additional source of heterogeneity. To partially account for this factor, rehabilitation-related differences were included post hoc as a covariate in sensitivity analyses. Nevertheless, the potential confounding impact of non-unified rehabilitation cannot be fully ruled out. Future studies should prospectively implement and monitor standardised rehabilitation protocols to reduce this source of bias and strengthen the comparability of outcomes.

Furthermore, although all patients were assessed at two follow-up points (8 and 12+ months), the overall follow-up period remains limited to medium-term recovery. Long-term consequences such as post-traumatic osteoarthritis, delayed vascular complications, or persistent muscle dysfunction could not be evaluated within the current timeframe.

Importantly, no correction for multiple testing (e.g., Bonferroni or Benjamini–Hochberg) was applied to the correlation analyses. Since these were exploratory, the reported *p*-values should be interpreted with caution due to the increased risk of Type I error.

Finally, while persistent neurovascular impairments were observed, it is important to recognise that recovery outcomes may also be influenced by factors not captured in this study, such as adherence to rehabilitation protocols, individual variations in healing capacity, and comorbid conditions. Future research should aim to integrate these clinical and behavioural factors to develop a more comprehensive understanding of long-term functional recovery.

## 7. Conclusions

The conducted study confirmed that the use of the Kocher–Langenbeck posterior surgical approach in the osteosynthesis of posterior wall acetabular fractures—despite its traditional recognition as the “gold standard”—is associated with pronounced and persistent impairments in both microcirculation and neuromuscular conduction. These findings are of fundamental importance, as they shift the focus from classical clinical and radiological parameters towards objective functional and haemodynamic indicators, thereby enabling a more comprehensive understanding of the consequences of trauma and surgical intervention.

The study’s methodology, which incorporated electromyography, chronaximetry, and rheovasography, allowed for the quantitative registration of impairments that had previously remained obscured by conventional assessment scales. The observed asymmetry of regional blood flow (exceeding 50% in certain segments), delayed recovery of peroneal nerve conduction, and significant deficits in the bioelectrical activity of the gluteal and quadriceps muscles convincingly demonstrate that surgical stabilisation alone does not guarantee full functional recovery. Moreover, the identified alterations confirm the multifactorial nature of complications, in which the condition of neurovascular structures plays a key role alongside mechanical fixation and quality of fracture reduction.

Thus, this work not only reaffirmed the relevance of assessing neuromuscular and vascular outcomes but also demonstrated the necessity of integrating such monitoring techniques into standard postoperative follow-up protocols. This is particularly crucial given that traditional markers of success—anatomical reduction and stable fixation—do not always correlate with patient quality of life and functional rehabilitation outcomes.

A comparison of the present findings with previous research further substantiates and extends existing knowledge on the outcomes of surgical treatment for acetabular fractures. Most authors—including Matta, Giannoudis, Negrin, and others—have emphasised the paramount importance of achieving accurate anatomical reduction to ensure favourable clinical results. Nevertheless, even with optimal fixation, the risk of degenerative complications, neurological deficits, and post-traumatic osteoarthritis persists. The current results complement this understanding by demonstrating that long-term functional insufficiency is also influenced by microcirculatory disturbances and peripheral nerve conduction deficits, as evidenced by rheovasographic and electromyographic assessments.

Particular significance lies in the differences observed among various muscle groups. The gluteus maximus and quadriceps exhibited the highest degree of asymmetry in bioelectrical activity, which may be attributed both to the severity of the initial trauma and to iatrogenic factors associated with surgical exposure. These observations are consistent with the findings of Borrelli et al., who reported that weakness of the gluteal and femoral muscles directly affects the extent of functional rehabilitation in patients. At the same time, the present findings indicate the presence of deeper pathophysiological mechanisms—not limited to muscular weakness but also involving persistent impairments in neural conduction and regional perfusion, which collectively hinder full recovery.

The clinical relevance of this study lies in the fact that the observed impairments may serve as predictors for the development of secondary complications, such as trophic disorders, degenerative joint changes, and post-traumatic coxarthrosis. This underscores the need to expand standard rehabilitation protocols by incorporating interventions aimed at restoring neuromuscular conduction and microcirculation (e.g., electrical stimulation, physiotherapy, and targeted strength training programmes). In the future, these findings may form the basis for the development of individualised rehabilitation strategies tailored to the specific neuromuscular impairments of each patient.

Despite the compelling results, this study has several limitations that must be taken into account when interpreting the data. Firstly, the limited sample size (34 patients) reduces the statistical power and restricts the generalisability of the findings. Conducting multicentre studies with a larger patient cohort would enhance the reliability and reproducibility of the results.

Secondly, the study had a retrospective design. Although the objective assessment methods used (rheovasography, chronaximetry, and electromyography) are highly sensitive, the absence of randomisation and prospective data collection does not fully eliminate the influence of confounding factors, such as individual differences in healing potential, rehabilitation quality, or comorbidities.

Thirdly, follow-up was limited to one year postoperatively. However, it is often in the later stages that complications such as progressive coxarthrosis or persistent contractures become evident. A longer follow-up period (at least 3–5 years) would provide a more comprehensive understanding of the pathogenesis of long-term functional impairments.

Nonetheless, despite these limitations, the present study provides an important contribution to understanding the multifactorial nature of outcomes following surgical treatment of acetabular fractures. It moves beyond traditional analyses focused on reduction accuracy and complication rates, offering instead a more comprehensive evaluation of microcirculatory function and neuromuscular conduction.

Future research should aim to implement prospective, randomised studies with larger cohorts and extended follow-up periods. Moreover, the application of advanced diagnostic technologies—such as functional MRI, ultrasound-based microcirculatory assessment, and neuroimaging techniques—may provide objective validation of the current findings. The integration of artificial intelligence and machine-learning algorithms could further enhance the prediction of postoperative complication risks by combining clinical, radiological, and functional parameters. Additionally, the design and clinical testing of personalised rehabilitation protocols incorporating neuromuscular stimulation and interventions to improve regional blood flow may substantially enhance functional recovery.

In conclusion, this study establishes a foundation for shifting from a purely mechanical or structural evaluation of outcomes to a more integrative framework that encompasses clinical, neurophysiological, and vascular dimensions. This conceptual transition opens new avenues for optimising both surgical management and rehabilitation strategies in patients with acetabular fractures.

## Figures and Tables

**Table 1:** Comparative Analysis of Rheovasographic Parameters and Asymmetry Coefficients (AC) of Blood Flow in the Lower Limb at 8 Months and after 12 Months Following Open Reduction and Internal Fixation of Posterior Acetabular Wall Fractures via the Kocher–Langenbeck Approach.

Group	Segment	A2			DCI			DSI			BF		
		>	<	AC%	>	<	AC%	>	<	AC%	>	<	AC%
8-month group	Thigh	0.012	0.01	30%	59	48	28%	50	41	26%	11	9	19%
	Shin	0.010	0.03	58%	46	39	21%	46	36	30%	11	10	12%
	Foot	0.090	0.08	31%	49	36	51%	51	38	47%	10	9	19%
12-month and later group	Thigh	0.014	0.011	33%	61	47	43%	48	35	38%	11	9	26%
	Shin	0.050	0.04	46%	49	38	35%	45	33	45%	11	10	14%
	Foot	0.100	0.08	50%	50	35	50%	53	41	38%	10	9	12%

Legend of abbreviations: A2—Amplitude of the systolic wave (mL); DCI—Dicrotic Index (%); DSI—Diastolic Index (%); BF—Blood Filling (in mm); >—Value on the unaffected limb; <—Value on the operated limb; AC%—Asymmetry Coefficient (%).

**Table 2:** Comparative Analysis of Rheovasographic Parameters (RVG) of the Thigh in Patients More Than 12 Months after Open Reduction and Internal Fixation of Posterior Acetabular Wall Fractures via the Kocher–Langenbeck Approach.

RVG Parameters	Limb	Mean Values	Std. Dev.	Std. Error
Pulse blood filling (A_2_)	Healthy	0.014	0.007	0.002
	Operated	0.011	0.005	0.001
	α/β/AC	0.757/AC = 24.3%	0.104	0.027
Arterial capillary index (DCI)	Healthy	63.730	15.673	4.047
	Operated	47.600	14.965	3.864
	α/β/AC	0.725/AC = 27.5%	0.169	0.044
Venous capillary index (DSI)	Healthy	47.800	13.991	3.612
	Operated	35.067	9.830	2.538
	α/β/AC	0.732/AC = 26.8%	0.133	0.034
Rheographic index (RI)	Healthy	10.933	2.017	0.521
	Operated	8.800	1.373	0.355
	α/β/AC	0.819/AC = 18.1%	0.124	0.032

Legend of abbreviations: α—Value on operated limb; β—Value on healthy limb; AC—Asymmetry coefficient.

**Table 3:** Chronaximetry Parameters of the Peroneal and Tibial Nerves in Patients after Open Reduction and Internal Fixation of Posterior Acetabular Wall Fractures via the Kocher–Langenbeck Approach.

Number of Patients: 34	8 Months Follow-Up						12 Months and Later					
	Nerves											
	Peroneal			Tibial			Peroneal			Tibial		
	>	<	AC%	>	<	AC%	>	<	AC%	>	<	AC%
Total values	344	299	885	408	308	466	250	186	660	312	244	399
Mean values	23	15	59%	27	21	31%	17	12	44%	21	16	27%

Legend of abbreviations: >—value on the healthy limb; <—value on the operated limb; AC%—asymmetry coefficient.

**Table 4:** Electromyographic (EMG) Parameters of Lower Limb Muscles in Patients One Year after Open Reduction and Internal Fixation of Posterior Acetabular Wall Fractures via the Kocher–Langenbeck Approach.

Muscles	Limb/AC	Mean Signal Value (mV)	Standard Deviation	Standard Error
Paravertebral muscles	Healthy	42.33	10.998	2.840
	Operated	31.0	11.680	3.016
	α/β/AC	0.733/AC = 26.7%	0.183	0.047
Gluteus maximus	Healthy	42.0	10.823	2.795
	Operated	25.333	6.399	1.652
	α/β/AC	0.61/AC = 39%	0.108	0.028
Biceps femoris	Healthy	45.0	9.820	2.535
	Operated	30.667	10.499	2.711
	α/β/AC	0.677/AC = 32.3%	0.149	0.038
Rectus femoris	Healthy	49.0	9.856	2.545
	Operated	31.333	7.898	2.039
	α/β/AC	0.642/AC = 35.8%	0.099	0.025
Tibialis group	Healthy	57.0	13.202	3.409
	Operated	36.0	18.048	4.660
	α/β/AC	0.627/AC = 37.3%	0.285	0.074
Peroneal group	Healthy	47.333	10.834	2.797
	Operated	30.0	14.015	3.619
	α/β/AC	0.641/AC = 35.9%	0.289	0.075
Gastrocnemius	Healthy	52.667	12.081	3.119
	Operated	37.0	12.071	3.117
	α/β/AC	0.698/AC = 30.2%	0.158	0.041

Legend of abbreviations: α—Value on operated limb; β—Value on healthy limb; AC—Asymmetry coefficient.

## Data Availability

The data supporting the findings of this study are available from the corresponding author upon reasonable request. The data are not publicly available due to privacy and ethical restrictions.

## Abbreviations

The following abbreviations are used in this manuscript:

AC	Asymmetry Coefficient
AI	Artificial Intelligence
AIP	Anterior Intrapelvic Approach (Stoppa approach)
A_2_	Systolic Wave Amplitude (Reovasographic Parameter)
BF	Blood Flow
CT	Computed Tomography
DCI	Dicrotic Index
DSI	Diastolic Index
EMG	Electromyography
IRB	Institutional Review Board
MRI	Magnetic Resonance Imaging
RI	Rheographic Index
RVG	Rheovasography
